# Bioelectrical Impedance Analysis Demonstrates Reliable Agreement with Dual-Energy X-ray Absorptiometry in Identifying Reduced Skeletal Muscle Mass in Patients with Metabolic Dysfunction-Associated Steatotic Liver Disease and Hypertension

**DOI:** 10.3390/diagnostics14202301

**Published:** 2024-10-16

**Authors:** Anna F. Sheptulina, Ekaterina O. Lyusina, Elvira M. Mamutova, Adel A. Yafarova, Anton R. Kiselev, Oxana M. Drapkina

**Affiliations:** 1 Department of Fundamental and Applied Aspects of Obesity, National Medical Research Center for Therapy and Preventive Medicine, 101990 Moscow, Russia; 2 Department of Therapy and Preventive Medicine, A.I. Evdokimov Moscow State University of Medicine and Dentistry, 127473 Moscow, Russia; 3 Coordinating Center for Fundamental Research, National Medical Research Center for Therapy and Preventive Medicine, 101990 Moscow, Russia

**Keywords:** body composition, fat mass, hypertension, metabolic dysfunction-associated steatotic liver disease, skeletal muscle mass

## Abstract

**Background/Objectives:** Body composition (BC) affects the risk of developing metabolic dysfunction-associated steatotic liver disease (MASLD) and hypertension (HTN). Currently, dual-energy X-ray absorptiometry (DEXA) is considered the gold standard for assessing BC, even though it has some limitations, including immobility, ionizing radiation, and patient weight restrictions. The aim of the study was to evaluate the correlations of BC parameters measured by bioelectrical impedance analysis (BIA) with those measured by DEXA in patients with MASLD and HTN. **Methods:** Overall, 78 patients with MASLD and HTN underwent the following study procedures: compilation of an anamnesis, physical examination of a patient, laboratory tests, abdominal ultrasound, BIA, DEXA, and anthropometric measurements. **Results:** The agreement between BIA and DEXA in diagnosing reduced skeletal muscle mass (SMM) in patients with MASLD and HTN was moderate (kappa values were 0.440 and 0.404 in males and females, respectively). Significant strong direct correlations were found between fat mass (FM) and body fat percentage measured by BIA with corresponding measurements by DEXA (*p* < 0.001 for both). The area under the receiver operating characteristic curves (AUC) of SMM to body weight ratios calculated using BIA data were 0.834 and 0.929 for reduced appendicular SMM determined by DEXA in males and females with MASLD and HTN, respectively. **Conclusions:** In conclusion, BIA is an easy-to-use and widely available tool for assessing SMM and FM in patients with MASLD and HTN, demonstrating reliable agreement with DEXA measurement results and completely free of its limitations.

## 1. Introduction

Metabolic dysfunction-associated steatotic liver disease (MASLD) is among the leading causes of liver dysfunction worldwide. The prevalence of MASLD is increasing globally: European and American researchers report its rates at the level of up to 40%, while about 30% of population in Asian countries suffer from this disease [1,2]. According to Wong et al., the prevalence of MASLD in Middle East and North Africa reaches 37%, while in the Asia–Pacific region it ranges from 25% to 28% depending on the diagnostic tool used to establish the diagnosis of this liver disease (computed tomography vs. abdominal ultrasound, respectively) [3]. In Russia, the prevalence of MASLD is similar to the global average, and according to the multicenter observational study DIREG2, 37.3% of patients seeking outpatient care were diagnosed with MASLD [4].

Currently, MASLD is considered a multisystem disorder associated with cardiovascular diseases (CVD) [5], T2D [6], cholelithiasis [7], chronic kidney disease [8], obstructive sleep apnea syndrome [9], and obesity [10]. The current mainstay of treatment for MASLD, which can improve patient outcomes and prognosis, is lifestyle modification via increasing physical activity and changing the diet. Body composition in patients with MASLD, in particular the amount of muscle mass, plays an important role in the choice of lifestyle modification strategy. It is well established that the calculation of body mass index (BMI) and the measurement of waist circumference (WC), which are widely used for diagnosing obesity and for assessing the metabolic risks, do not provide any information concerning skeletal muscle mass (SMM) [11,12]. However, in a study by Kim et al. including patients with and without MASLD who were followed for seven years, it was established that increasing SMM could potentially decelerate, stop, or even reverse the progression of MASLD over time and help resolve existing MASLD [13]. Thus, the use of tools allowing for the assessment of the amount of skeletal muscle in these patients in routine clinical practice is of critical importance. Such methods include bioelectrical impedance analysis (BIA), dual-energy X-ray absorptiometry (DEXA), computed tomography (CT), and magnetic resonance imaging (MRI) [14,15].

Despite their accuracy, MRI and CT are rarely used in primary healthcare settings for the diagnosis of reduced SMM for the following reasons: high equipment cost; large, stationary equipment, which makes them unsuitable for use in settings that require mobility, such as bedside or home-based care; the need for highly skilled personnel; and undefined threshold values (there are no established threshold values for identifying low muscle mass when using these diagnostic methods) [16].

DEXA is considered the gold standard for assessing body composition for several reasons. These include the ability to measure regional body composition by subdividing the body using certain well-defined cut lines, along with the accuracy and stability of this method. However, DEXA has some limitations as well: it is immobile, has a weight restriction of 130 kg, and exposes the patient and operator to ionizing radiation, although the dose for both is very small [17].

In turn, BIA is a noninvasive, inexpensive, and widely used approach to measuring body composition and assessing clinical status. The main advantage of BIA over other methods of body composition assessment is the almost complete absence of contraindications to its use. In addition, BIA allows for rapid assessment of body composition [18]. At the same time, BIA is generally thought to provide less accurate results on body composition compared to radiological methods. This may be due to the fact that it relies on a large number of prediction equations using linear regression to estimate body composition based on many predefined variables. The shortcoming is that the latter may vary across populations and are obtained from healthy individuals [19]. However, BIA allows for the analysis of several additional parameters, such as phase angle (PhA) and body cell mass percentage (BCMP), which reflect functional status, overall physical performance, and metabolic activity. In addition, it is portable, easy to use, and safe [20]. In addition, few previous studies have reported good concordance between DEXA and BIA for the assessment of body composition in overweight and obese subjects [21,22].

Thus, taking all the aforesaid into consideration the main hypothesis for this research was formulated the following: a noninvasive, inexpensive method of BIA is as accurate as DEXA for diagnosing reduced SMM in patients with MASLD and HTN.

## 2. Materials and Methods

### 2.1. Study Participants

Our cross-sectional single-center trial included consecutive patients with HTN and MASLD, aged 20 to 70 years, who met the inclusion criteria and were treated at the Department of Clinical Diagnostics, the National Medical Research Center for Therapy and Preventive Medicine of the Russian Federation Ministry of Healthcare, from February 2023 through April 2024. The study protocol was approved by the Research Ethics Committee of the institution (protocol No. 08-03/22 of 27 December 2022). All included patients signed the informed consent form.

The inclusion criteria were as follows: (1) age from 18 to 70 years; (2) confirmed diagnosis of MASLD; (3) confirmed diagnosis of HTN; and (4) the absence of concomitant liver disease (based on serological testing for viral hepatitis B and C, as well as the level of γ-globulin in serum, the content of immunoglobulin G, and the serum concentration of ferritin).

Exclusion criteria comprised concomitant liver disease, alcohol abuse (defined as a score ≥ 8 based on the Russian version of the Alcohol Use Disorders Identification Test, RUS-AUDIT), mental disorders, acute infectious diseases, or exacerbation of chronic noncommunicable diseases (within four weeks prior to inclusion), oncology without radical treatment, undifferentiated connective tissue disease, severe cardiac arrhythmias, uncontrolled HTN, history of acute cerebrovascular accident, inflammatory bowel disease, type 1 diabetes mellitus or T2D, hypoparathyroidism, chronic kidney disease requiring dialysis, pregnancy and breastfeeding, lower limb fractures within 6 months prior to the study with ongoing negative impact on functional status, any clinically significant disorders or diseases that impair mobility and self-care, and also the absence of a signed consent form.

All patients underwent examination according to the study protocol, which included assessment of disease duration and use of concomitant medications. Study procedures were performed during a single visit to the center. These included compilation of an anamnesis, physical examination of a patient (assessing for the presence of peripheral edema, and conducting lung auscultation, as well as percussion and palpation of the abdomen), laboratory tests, abdominal ultrasound test, point shear-wave elastography of the liver (Philips Affiniti 70, Philips, Amsterdam, the Netherlands), BIA, DEXA, and the following anthropometric measurements: weight, height, BMI, waist circumference (WC), and hip circumference (HC).

### 2.2. Diagnosing Metabolic Dysfunction-Associated Steatotic Liver Disease and Hypertension

MASLD was diagnosed based on the criteria proposed by the 2023 Multisociety Delphi Consensus Statement on New Fatty Liver Disease Nomenclature, including at least one cardiometabolic risk factor and the presence of signs of hepatic steatosis detected by abdominal ultrasound, in particular liver–kidney contrast, liver parenchymal echogenicity, reduced hepatic vasculature or impaired visualization of the deeper liver parenchyma and diaphragm [23].

In addition, to assess the likelihood of hepatic steatosis, the fatty liver index (FLI) was calculated as follows [24]:

FLI = [e^0.953 × ln (TG) + 0.139 × BMI + 0.718 × ln (GGT) + 0.053 × WC − 15.745^)/(1 + e^0.953 ln (TG) + 0.139 × BMI + 0.718 × ln (GGT) + 0.053 × WC − 15.745^] × 100,

where TG stands for triglycerides and GGT is an acronym for gamma-glutamyl transferase.

An FLI value less than 30 (negative likelihood ratio = 0.2) allowed us to exclude hepatic steatosis, whereas an FLI value of 60 or more (positive likelihood ratio = 4.3) confirmed the presence of hepatic steatosis [24].

We also performed liver stiffness measurement using point shear-wave elastography. At least ten valid measurements were included in the final report. We considered liver stiffness assessment reliable if the following criteria were met: 10 valid measurements, success rate > 60%, and the ratio of interquartile range to median (IQR/M) ≤ 30% [25,26].

The diagnosis of HTN was established based on the patient’s medical history and provided medical documentation (including data on the exclusion of secondary HTN). HTN was classified as follows: grade 1 HTN with systolic blood pressure (SBP) values of 140–159 mmHg and/or diastolic blood pressure (DBP) values of 90–99 mmHg; grade 2 HTN with SBP values of 160–179 mmHg and/or DBP values of 100–109 mmHg; and grade 3 HTN with SBP values ≥ 180 mmHg and/or DBP values ≥ 110 mm mmHg [27].

### 2.3. Anthropometric Measurements

Height was measured using a R-St-MSK (MSK-234) stadiometer (Medstalkonstruktsiya LLC, Ufa, Russia) using the standard technique [28]. Weight was determined using VMEN-200-50/100-I-ST-A electronic medical floor scales (TVES LLC, Tambov Oblast, Tambov, Russia) placed on a flat smooth floor. Patients stood on the scales in light clothing, without shoes, distributing their weight evenly on both feet and without holding on to surrounding objects. Three consecutive measurements were taken until similar values were obtained. BMI was calculated by dividing weight (kg) by height in meters squared (m^2^) [29].

WC and HC were measured with the patient standing, abdomen relaxed, arms hanging freely at the sides, and heels together. WC was measured with a tape measure at the narrowest part of the abdomen, at the level of the natural waist. The measurement was taken at the end of a normal exhalation, ensuring that the tape was pressed against the clothing without pressing into the skin. HC was measured by placing the tape around the hips at the level of maximum protrusion of the buttocks [29].

### 2.4. Bioelectrical Impedance Analysis

BIA was performed using the ABC-02 MEDAS analyzer (MEDAS Science and Technology Center LLC, Moscow, Russia). The ABC-02 MEDAS analyzer with the Russian abbreviation “ABC” meaning “analyzer of water compartments”, represents a phase-sensitive two-frequency lead-type bioelectrical impedance instrument generating a low-intensity (800 μA) electrical current and utilizing mainly a tetrapolar electrode configuration. With an ABC-02 MEDAS analyzer, body composition was evaluated using well-known published equations, such as for fat-free mass (FFM) in adults aged 17–62 years with 3–56% body fat [30] and extracellular water in adults [31].

BIA of body composition was performed after a few minutes of patients staying indoors, with minimal clothing, on the right side of the body in the lying position with arms and legs slightly abducted according to a conventional tetrapolar scheme at the electrical current frequencies 5 and 50 kHz. Disposable bioadhesive F9049 electrodes (FIAB, Vicchio, Florence, Tuscany, Italy) were used. Patients were informed of the necessary preparation before the procedure: the last meal should be at least 4 h before the study, diuretics should be discontinued 5 days before the procedure, and caffeinated beverages should be avoided for 1 day before the study.

The following BIA parameters were included in the analysis: lean mass (LM), SMM, SMM to body weight ratio (SMM/W), fat mass (FM), body fat percentage (%BF), BCMP, and PhA. The latter was calculated at 50 kHz as follows [28]:

PhA = atan (Xc/R) × 180°/π,

where Xc is the reactance, and R—the resistance.

The model to estimate skeletal muscle mass was as follows [32]:

SMM = 0.401 × (H2/R) + 3.825 × Sex−0.071 × Age + 5.102,

where H is height, and R—the resistance.

The following correction was applied to SMM before the calculation of SMM/W ratio, which reduced the error in the assessment of SMM by BIA in subjects with BMI ≥ 30 kg/m^2^ from 1.63 ± 2.40 to 0.01 ± 2.11 kg [33]:

SMM_BIA, corrected_ = SMM_BIA, uncorrected_−0.298 × (BMI−30 kg/m^2^),

where SMMBIA, corrected is skeletal muscle mass measured by BIA after the application of correction; SMMBIA, uncorrected—skeletal muscle mass measured by BIA before the application of correction, and BMI—body mass index.

For convenience and brevity, we further denoted SMM_BIA, corrected_ as SMM.

A reduction in the SMM/W ratio was determined based on a decrease in this parameter for each individual patient below the percentage of the mean normal value for the general Russian population, as surveyed in Russian Health Centers in 2010–2012. The SMM/W threshold value for defining reduced skeletal muscle mass was <31.5% for men and <22.1% for women [34].

The bioelectrical impedance (BI) instrument ABC-02 MEDAS provides stable measurements with a relative error value of about 0.1% for R and 1% for Xc [28]. Quality control of BIA measurements using the ABC-02 MEDAS device was ensured by initial training of personnel on the analyzer from the manufacturer, as well as by conducting a training session organized by the manufacturer before the start of the study. In addition, to check the performance of the BI instrument itself, we measured the supplied equivalent electrical circuit daily before the start of patient measurements to ensure minimal differences between measured and expected values of the resistance and the reactance, respectively. It is worth noting that for the F9049 electrodes used in the present research, minimal inter-electrode differences in BIA parameters of 1.30 ohm R, 0.26 ohm Xc, 0.02° PhA, and 0.04% percentage body fat have been previously described [28].

### 2.5. Dual-Energy X-ray Absorptiometry

Densitometry was performed using a Lunar iDXA instrument, and soft tissue composition was estimated using the enCORE software platform, version 18.0 (General Electric HealthCare, Chicago, IL, USA). The following DEXA parameters were recorded: SMM; appendicular skeletal muscle mass, i.e., SMM from both legs and arms (ASMM); ASMM to body weight ratio (ASMM/W); and appendicular skeletal muscle mass index (ASMI, kg/m^2^) [33]. The ASMI threshold value for defining reduced SMM was ≤7.26 kg/m^2^ for men and ≤5.50 kg/m^2^ for women [15]. For the ASMM/W ratio, the threshold value for diagnosing reduced SMM was <28.27% for men and <23.47% for women [35]. In addition, visceral and subcutaneous fat mass, FM, and %BF were measured.

### 2.6. Statistical Analyses

The Kolmogorov–Smirnov test was used to analyze the type of distributions. Nonparametric tests were performed because the obtained data were not normally distributed. Continuous data were expressed as medians and interquartile ranges (IQR: 25th–75th percentiles), while categorical variables were presented as the number of events (%). Spearman’s rank correlation coefficient (ρ) was used to analyze the strength of the association between two variables. The strength of the correlation was assessed according to the Chaddock scale as negligible (0.1 ≤ ρ < 0.3), weak (0.3 ≤ ρ < 0.5), moderate (0.5 ≤ ρ < 0.7), strong (0.7 ≤ ρ < 0.9), or very strong (0.9 ≤ ρ < 1.0) [36]. The nonparametric Kruskal–Wallis test was used for multiple comparisons. Comparisons between two groups were performed using the Mann–Whitney U test. Pearson’s chi-squared test was employed to compare the number of events between the patient groups. Cohen’s kappa statistic (κ) was used to assess the agreement between the muscle mass data obtained by BIA and DEXA. The diagnostic value of SMM/W ratio calculated from the BIA results to exclude a reduced ASMM/W ratio determined by DEXA was estimated by the area under the receiver operating characteristic curve (AUC). Optimal threshold values for the SMM/W ratio were identified at the maximum of overall sensitivity and specificity. Positive and negative predictive values (PPV and NPV, respectively) were calculated for the corresponding threshold values. Results with a two-sided *p* < 0.05 were considered statistically significant. Data were analyzed using IBM SPSS statistical software, version 27.0 (IBM Corp., Armonk, NY, USA).

## 3. Results

### 3.1. Patient Characteristics

From February 2023 through April 2024, we examined 88 patients. Of these, 78 patients met the inclusion criteria, while 10 patients were excluded from the final analysis for the following reasons: 2 patients tested positive for hepatitis C (HCV Ab positive), 1 patient tested positive for hepatitis B (positive HBsAg test result), 2 patients had a RUS-AUDIT score of 8 or more; 1 patient had incomplete recovery of the right hand function after a radius fracture that occurred 3 months before inclusion; 1 patient developed an attack of gout the day before the scheduled visit; 2 patients had focal lesions of presumed malignancy detected during ultrasound examination of the abdominal organs: 1 patient had a kidney tumor, another had a tumor of the head of the pancreas; finally, 1 patient did not show up for the scheduled visit and did not respond to phone calls and emails. Thus, 78 patients with MASLD and HTN who were eligible were included in the final analysis. Their main characteristics are shown in Table 1.

The median age of the patients was 58 years (IQR 51–65); 48 (61.5%) were women. Most patients (74.5%) were obese, and only 2 patients had a normal BMI. Both of these patients were women aged 61 and 62 years with a WC of 94 cm and 91 cm, respectively. According to BIA, both women had excess body fat (26.3 kg and 23.5 kg, correspondingly), which accounted for 38% and 39% of their body weight, respectively. Thus, despite normal BMI values, these patients were considered obese. Changes in glycemic status were observed in 21 patients (26.9%), including 8 patients (10.3% of the total number of patients) with T2D and 13 patients (61.9%) with impaired fasting glycemia. According to the inclusion criteria, all patients had elevated blood pressure, including 29.4% (*n* = 23) with grade 1 HTN, 34.6% (*n* = 27) with grade 2 HTN, and 36% (*n* = 28) with grade 3 HTN. Elevated total cholesterol was detected in 60 patients (77%), while the median cholesterol level was 197.2 mg/dl (IQR 224.3–239.7).

Analyzing the data presented in Table 1, we should emphasize that there was no significant biochemical activity: the median ALT and AST values (IQR) were 23 (15.8–38.5) and 22 (18–26) IU/l, respectively, while ALT values exceeding the upper limit of normal range (34 IU/l) were detected in only 9 patients (11.5%). In addition, no significant liver fibrosis was identified; the median liver stiffness was 4.1 (5.4–6.4) kPa, and just 9 patients (11.5%) had liver stiffness exceeding 7 kPa.

Table 2 and Table 3 present the results of body composition assessment using BIA and DEXA, respectively. Absolute (kg) and relative (%) body fat measured by BIA exceeded normal values in all included patients, with medians of 34.3 kg and 38.3%, respectively. These results indicate the presence of obesity in all patients of the study group, regardless of BMI values.

According to BIA results, only one 66-year-old man (1.3%) had reduced SMM, while decreased SMM/W index values were recorded in 12 patients (15.4%): 5 men and 7 women with a median age of 59.5 (53.8–67.8) years. All these patients were obese.

PhA value < 5.4 was detected in 5 patients (6.4%), indicating the presence of low physical activity. All these study subjects were women aged 60 to 69 years with overweight or obesity (based on BMI values). Reduced BCMP was recorded in 6 patients (7.7%) (including 5 women). Their median age was 64.5 (59.3–66) years.

DEXA results are presented in Table 3. Decreased ASMM/W index values were found in 5 male patients (16% of the total number of male patients included in the study, *n* = 30; median age: 66 (44–67) years) and 17 female patients (35.4% of the total number of female patients included in the study, *n* = 48; median age: 62 (58–66) years). ASMI index values were within reference ranges in all patients with MASLD and HTN included in our study.

### 3.2. Correlations of Body Composition Parameters Measured by BIA with Body Composition Parameters Measured by DEXA

Overall, parameters characterizing body fat content (FM, %BF) and skeletal muscle amount (SMM, SMM/W) measured by BIA demonstrated statistically significant correlation with similar parameters measured by DEXA (with the strength of the correlation varying from moderate to strong) both in male and female patients with MASLD and HTN (Table 4). Based on the kappa statistic, the agreement between BIA and DEXA in diagnosing reduced SMM in patients with MASLD and HTN was moderate (κ = 0.440 in men and κ = 0.404 in women).

Also, we observed statistically significant correlations of PhA with FM (ρ = −0.260, *p* = 0.025), and %BF measured by DEXA (ρ = −0.531, *p* < 0.001), as well as with SMM and indices characterizing the amount of SMM in the body measured by DEXA (SMM: ρ = 0.496, *p* < 0.001; ASMI: ρ = 0.472, *p* < 0.001; ASMM/W: ρ = 0.468, *p* < 0.001). Additionally, BCMP correlated with the following body composition parameters measured by DEXA: ASMI (ρ = 0.459, *p* < 0.001), %BF (ρ = −0.540, *p* < 0.001), FM (ρ = −0.260, *p* = 0.025), SMM (ρ = 0.516, *p* < 0.001), and ASMM/W index values (ρ = 0.485, *p* < 0.001).

**Figure 1:**
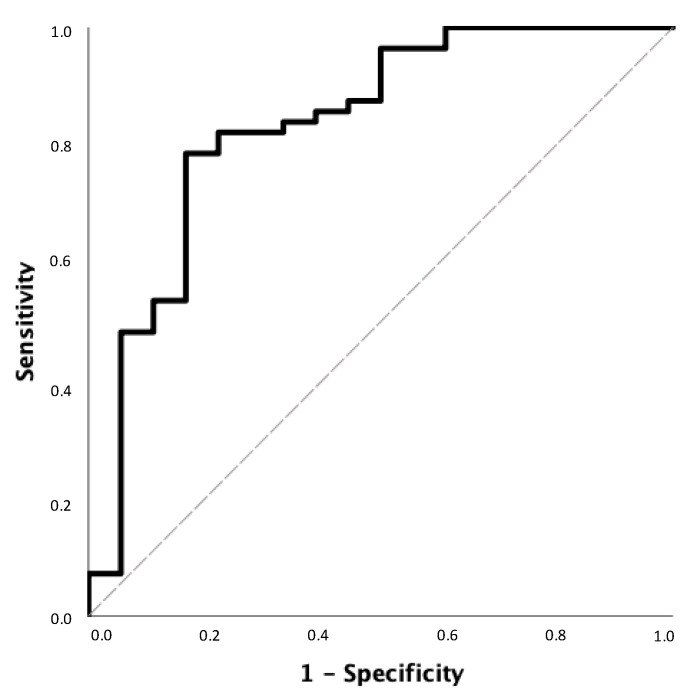
Receiver operating characteristic (ROC) curve for SMM/W ratio to exclude decreased ASMM/W index values measured by DEXA in female patients with MASLD and HTN. The area under the ROC curve (AUC) ± standard errors (SE) for SMM/W ratio was 0.834 ± 0.061; 95% confidence interval (CI): 0.715–0.954.

**Figure 2:**
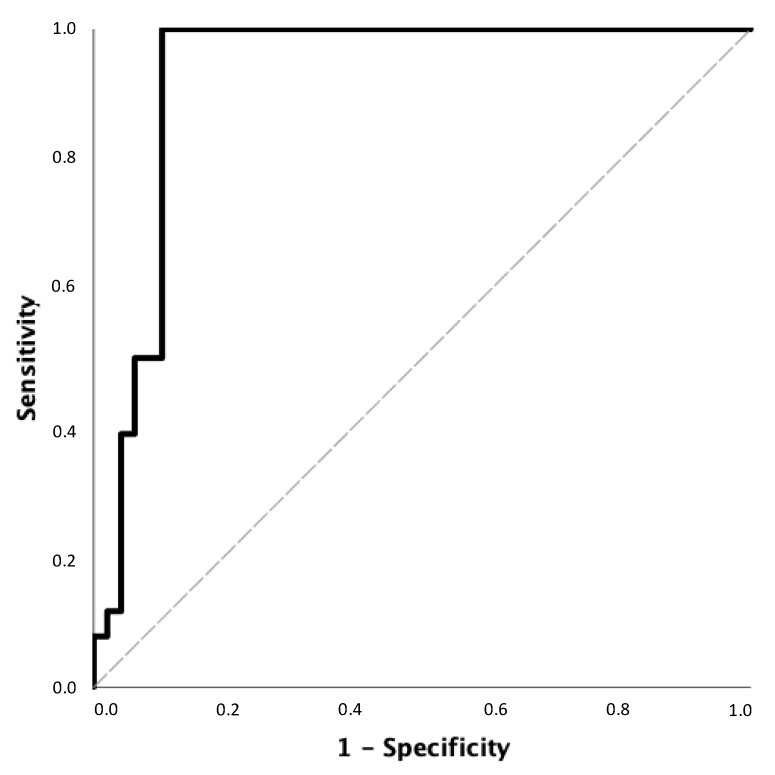
Receiver operating characteristic (ROC) curve for SMM/W ratio to confirm the presence of decreased ASMM/W index values measured by DEXA in male patients with MASLD and HTN. The area under the ROC curve (AUC) ± standard errors (SE) for SMM/W ratio was 0.929 ± 0.032; 95% CI: 0.865–0.992.

According to ROC analysis, SMM/W ratio < 25.3% could exclude the presence of reduced ASMM/W index values measured by DEXA in female patients with MASLD and HTN with a sensitivity of 78.6% and specificity of 83.3%. The NPV was 96.4% (Figure 1). In addition, an SMM/W ratio greater than 31.1% had a PPV for confirming the presence of decreased ASMM/W index values measured by DEXA in male patients with MASLD and HTN of 92.2% with a specificity of 89.6% and a sensitivity of 96.2% (Figure 2).

## 4. Discussion

Currently, much attention is paid to the issue of lifestyle modification as a method of prevention and treatment of patients with MASLD, which covers two main approaches: diet modification and increased physical activity. These approaches influence the body composition of patients with MASLD, in particular the amount of fat and skeletal muscles in the body, as well as their ratio.

Overweight and obesity are common in patients with MASLD and HTN [37]. These conditions are usually diagnosed with the calculation of BMI and/or measurement of WC. However, BMI and WC have several limitations, the most significant of which being that they do not provide information on the amount of fat and skeletal muscle in the body [11,38]. At the same time, according to the literature, it is SMM and skeletal muscle quality that determine the severity of liver steatosis, the risk of developing steatohepatitis, and the likelihood of MASLD resolution [13,39]. Moreover, fatigue, which is quite common among patients with MASLD and limits their ability to engage in physical activity, may be the result of low SMM and/or poor skeletal muscle quality due to fatty infiltration [40]. Thus, it seems reasonable to use special tools to assess the amount of fat and muscle in the body of patients with MASLD before providing any recommendations on lifestyle modification strategy in each specific case.

In this cross-sectional single-center study, body composition of 78 patients with MASLD and HTN was analyzed using two different methods, BIA and DEXA. According to our study results, a significant correlation was found between body composition parameters measured by BIA and those determined by DEXA in patients with MASLD and HTN. In addition, the agreement between BIA and DEXA in diagnosing reduced SMM in patients with MASLD and HTN was moderate (κ = 0.440 in men and κ = 0.404 in women). In support of these data, we demonstrated that the SMM/W ratio calculated based on BIA results strongly correlated with the ASMM/W ratio derived from DEXA in both men and women with MASLD and HTN (ρ = 0.609, *p* < 0.001 and ρ = 0.626, *p* < 0.001, respectively). In addition, the SMM/W ratio calculated from BIA results showed good to excellent AUCs for the detection of reduced ASMM/W index values in female (0.834 ± 0.061; 95% CI: 0.715–0.954) and male (0.929 ± 0.032; 95% CI: 0.865–0.992) patients with MASLD and HTN. These specific indices used to assess skeletal muscle mass in patients with MASLD and HTN in our study were considered by Cruz-Jentoft et al. [14,15] and Donini et al. [35] the most suitable and valid parameters to identify the relative reduction of muscle mass in patients with excess body fat. Moreover, the assessment of relative muscle mass using these specific indices, rather than absolute muscle mass, may help identify patients with MASLD and HTN who have an abnormal ratio of muscle to fat tissue in their bodies, even if their SMM measured either by BIA or DEXA is within the normal range of values. Indeed, it is currently well established that obesity may increase muscle mass because the body needs to move around excess weight [41,42], and the latter may exert a positive training stimulus on skeletal muscle.

The important issue associated with using BIA in obese patients, such as those with MASLD and HTN included in the present study, is an underestimation of FM and an overestimation of fat-free mass [43]. However, the overestimation of fat-free mass and SMM in patients with obesity may be minimized by the application of specific corrections, such as the correction for SMM developed by Jensen et al. [33] for subjects with a BMI ≥ 30 kg/m^2^, which we used in our study.

The results of the present study are in line with those obtained by Mainous et al., who analyzed body fat percentages among adults with a healthy BMI (18.5–24.9) and the association of this body composition parameter with the presence of MASLD. The authors showed that the prevalence of undiagnosed MASLD was significantly higher among adults with normal BMI who had an elevated body fat percentage and, consequently, lower SMM compared to those with normal BMI and a lower body fat percentage. They concluded that it is reasonable to use more informative body composition measures when screening for MASLD, thus emphasizing the limitations of BMI [44]. Similarly, in their study, Ariya et al. demonstrated that fat-free tissue was inversely and fat tissue was directly correlated with the risk of MASLD [45]. In addition, Zhang et al. indicated that non-obese healthy controls had lower body fat mass and lower body fat percentage, as well as lower waist–hip ratios compared to non-obese MASLD patients [46]. Taken together, these data demonstrate the importance of assessing body composition in patients with MASLD, including those with associated conditions, rather than measuring BMI or WC alone. Thus, the question is whether it is possible to use BIA instead of DEXA, which is regarded as the gold standard for body composition assessment, for the identification of reduced skeletal mass, as well as excess body fat in patients with MASLD, including those with MASLD and HTN.

To answer this question, Achamrah et al. conducted a study to compare body composition assessment by DXA and BIA according to the BMI in a large cohort of patients followed for malnutrition, obesity, or eating disorders in a nutrition unit from 2010 to 2016. Authors showed that whatever the BMI, BIA and DXA methods reported very large limits of agreement either for FM or fat-free mass [47]. Few previous studies have also reported good concordance between DEXA and BIA in overweight and obese subjects [21,22]. For instance, in their study of 28 subjects (10 men and 18 women) aged 20–60 years with BMI values ranging from 17.9 to 31.6 kg/m^2^, Stewart et al. found that measuring arm impedance is a simple method for assessing whole-body composition. The accuracy of this method was comparable to that of skinfold anthropometry and DEXA [21]. In the study by Thomson et al., which included 24 female patients with a BMI of 36.4 ± 4.3 kg/m^2^, the authors demonstrated that compared to DEXA, both multi-frequency and single frequency BIA accurately assessed changes in body composition with weight loss in female patients with overweight or obesity [22]. Moreover, body composition assessment via DEXA or BIA (as an alternative second choice) is recommended for the detection of sarcopenic obesity by the joint consensus statement of European Society for Clinical Nutrition and Metabolism (ESPEN) and the European Association for the Study of Obesity (EASO) [35].

It is worth noting that unlike DEXA, BIA allows for the measurement of two additional parameters that are characteristic of metabolic activity and overall physical performance: BCMP and PhA. We demonstrated that BCMP (i.e., the amount of metabolically active tissue in the body) and PhA calculated from BIA data were inversely related to the amount of adipose tissue measured by each method. BCMP is used as a marker of physical inactivity, as well as an indicator reflecting the basal metabolic rate. Meanwhile, PhA is a marker that evaluates the state of the body’s cells, the general level of physical performance, and the intensity of metabolism [48]. Therefore, the aforementioned data allow us to draw two main conclusions. First, the assessment of BCMP and PhA by BIA allows us to estimate the probability of physical inactivity in a patient based solely on this method, without additional instruments. Second, BCMP and PhA values are capable of predicting a reduction in SMM and a decrease in the basal metabolic rate, which are the key factors that hinder weight loss and an increase in physical performance. Both weight reduction and improved physical performance are decisive factors for the successful implementation of lifestyle modification strategies and the achievement of MASLD therapy goals.

These findings concerning BCMP and PhA obtained in our study are in line with those described by the following studies. For instance, Yamada et al. showed that subjects who engaged in regular training had significantly higher PhA values compared to those who did not perform any physical exercises [49]. In turn, Otsuka et al. suggested that PhA might reflect the quality of skeletal muscles, as it significantly correlated with the ratio of intermuscular adipose tissue to thigh muscle cross-sectional area [50]. As to BCMP, Guerrini et al. showed that body cell mass significantly correlated with the baseline activity of daily living [51]. Furthermore, Moonen et al. suggested that measuring body cell mass might be important for nutritional screening, as this parameter provides information regarding metabolic rate and protein requirements [52].

Our results have broad practical implications. We demonstrated reliable agreement between BIA and DEXA in detecting reduced SMM in patients with MASLD and HTN. DEXA is currently considered the gold standard for body composition assessment, although its performance can be challenging because the DEXA device is immobile, has a weight limit of 130 kg, uses ionizing radiation, and requires a trained operator. At the same time, BIA is a noninvasive, inexpensive, and easy-to-perform method with almost no contraindications for its use. It can be performed in both outpatient and inpatient settings. BIA provides an immediate and easy-to-interpret examination report, thereby supporting the physician’s decision regarding the further management of an individual patient with MASLD and HTN.

Given the importance of body composition, particularly SMM, in the development and progression of both MASLD and HTN, as well as in the choice of lifestyle modification strategies and their effectiveness, body composition assessment, rather than the measurement of WC and calculation of BMI alone, should be a critical component of the treatment and follow-up of patients with MASLD.

## 5. Strengths and Limitations

The strengths of our study include a comprehensive assessment of patient demographics, anthropometric characteristics, and liver disease severity in patients 20 to 70 years of age with MASLD and HTN. For the diagnosis of MASLD, we followed the recommendations of an expert consensus published in 2023 [14] and the guidelines of the European Association for the Study of the Liver [53]. The use of two-frequency BIA device allowed for the estimation of all body composition parameters except for the extracellular water at a frequency of 50 kHz, while the later was assessed at a frequency of 5 kHz. In order to minimize the error associated with SMM assessment using BIA in obese patients we used the correction for SMM developed by Jensen et al. [33]. To the best of our knowledge, this is the first study to demonstrate robust agreement between DEXA and BIA in assessing body composition in patients with MASLD and HTN without significant liver fibrosis.

This study has several limitations. First, we had a relatively small sample size, although the participants were a representative sample of well-characterized MASLD patients without significant liver fibrosis and with grades 1–3 HTN as a cardiometabolic risk factor. Second, we did not use liver biopsy to determine the activity of MASLD and liver fibrosis stage, since the recommendations of an expert consensus published in 2023 [23] and the guidelines of the European Association for the Study of the Liver [53] suggest that the diagnosis of MASLD can be established based on the identification of liver steatosis by liver ultrasound and the detection of one or more cardiometabolic risk factors in the patient, provided that other etiologies of liver steatosis are excluded.

## 6. Conclusions

The results of this study imply that the initial assessment of FM and SMM via BIA and/or DEXA, rather than only BMI calculation and WC measurement, may be useful for developing accurate and personalized lifestyle recommendations, thereby improving the prevention and treatment of MASLD and related conditions.

The BIA method has proven to be an easy-to-implement, widely available tool for assessing SMM and FM in patients with MASLD and HTN, demonstrating reliable agreement with DEXA and completely free of its limitations. Also, in addition to body composition analysis, BIA allows for the assessment of the functional status and overall physical performance of patients without the need for additional instruments.

## Figures and Tables

**Table 1:** Demographic, anthropometric, and biochemical parameters of 78 MASLD patients.

Parameter	Patients with MASLD and HTN (*n* = 78)	Normal Range	Patients Outside the Normal Range, *n* (%)
Gender: female, *n* (%)	48 (61.5)	-	-
Age, years	58 (51–65)	-	-
BMI, kg/m^2^	33.2 (30.1–36.8)	18.5–24.99	76 (97.4)
Normal weight, *n* (%)	2 (2.5)		
Overweight (25 kg/m^2^ ≤ BMI < 30 kg/m^2^), *n* (%)	18 (23)		
Obesity (BMI ≥ 30 kg/m^2^), *n* (%)	68 (74.5)		
Waist circumference, cm	107 (102–115)		
Waist circumference (male), cm	111 (106–117)	≤94 cm	30 (100)
Waist circumference (female), cm	106 (100–112.7)	≤80 cm	48 (100)
Hip circumference, cm	113 (109–119)		
Waist circumference/hip circumference ratio, (male)	1 (0.96–1)	≤0.95	25 (83.4)
Waist circumference/hip circumference ratio, (female)	0.9 (0.87–0.97)	≤0.8	47 (97.9)
Platelets, 10^9^/L; (normal range)	251 (209–286.3)	150–400	0 (0)
ALT, IU/L	23 (15.8–38.5)	0–34	9 (11.5)
AST, IU/L	22 (18–26)	0–34	6 (7.7)
GGT, IU/L	31.5 (21–45.3)	0–38	30 (38.5)
Total bilirubin, mg/dL	0.5 (0.7–0.9)	0.3–1.2	3 (3.8)
Glucose, mg/dL	97.2 (104.4–109.8)	73.9–106.3	30 (38.5)
Total cholesterol, mg/dL	197.2 (224.3–239.7)	<193.3	61 (78.2)
HDL, mg/dL	46.39 (57.99–65.72)		
HDL (male), mg/dL	46.4 (39.4–61.9)	>38.7	4 (13.4)
HDL (female), mg/dL	59.2 (50.3–65.7)	>46.4	6 (12.5)
Triglycerides, mg/dL	122.5 (87.5–175)	<150.5	28 (35.9)
Creatinine, mmol/L	67.5 (74.0–89.0)	51–98	6 (7.7)
Uric acid, µmol/L	327.2 (362.9–416.4)	154.7–356.9	33 (42.3)
Albumin, g/L; (normal range)	45 (43.0–47.0)	35–52	0 (0)
HOMA-IR > 2.7, *n* (%)	47 (70.1)	0.5–1.4	-
FLI	71 (85–93.5)	-	4 (5.1)
FLI ≥ 30, *n* (%)	74 (94.9)		
FLI ≥ 60, *n* (%)	64 (82.1)		
Liver stiffness, kPa (pSWE)	4.1 (5.4–6.4)	(<7)	9 (11.5)

Note: The presented values represent the frequency (%) or median (interquartile range); ALT, alanine aminotransferase; AST, aspartate aminotransferase; GGT, gamma-glutamyl transpeptidase; BMI, body mass index; HDL, high-density lipoprotein; HOMA-IR, homeostasis model assessment of insulin resistance; HTN, hypertension; FLI, fatty liver index; MASLD, metabolic dysfunction-associated steatotic liver disease; pSWE, point shear-wave elastography.

**Table 2:** Parameters of body composition based on bioelectrical impedance analysis.

Parameter	Value
SMM, kg	25.1 (20.5–35.9)
SMM (male), kg	36.3 (32.9–37.9)
SMM (female), kg	21.5 (19.6–24.1)
SMM/W (male), %	33.7 (32.8–34.8)
SMM/W (female), %	24.4 (22.5–26.9)
Lean mass, kg	56 (48.4–71.5)
Lean mass (male), kg	73.2 (68.7–76.6)
Lean mass (female), kg	50.2 (47.4–54.0)
Fat mass, kg	34.3 (27.8–40.7)
Fat mass (male), kg	32.4 (25.6–36.8)
Fat mass (female), kg	35.5 (28.7–45.7)
Percentage of body fat, %	38.3 (31.5–43.4)
Percentage of body fat (male), %	30.6 (28.9–33.4)
Percentage of body fat (female), %	42.3 (38.5–46.2)
BCMP, %	55.7 (52.4–58.7)
Phase angle	6.5 (5.9–7.1)

Note: The presented values represent the median (interquartile range); BCMP, body cell mass percentage; SMM, skeletal muscle mass; SMM/W: skeletal muscle mass to body weight ratio.

**Table 3:** Parameters of body composition based on dual-energy X-ray absorptiometry.

Parameter	Value
SMM, kg	44.7 (39–59.8)
SMM (male), kg	61.5 (57.8–64.4)
SMM (female), kg	39.6 (37.7–42.7)
ASMI, kg/m^2^	8.3 (7.6–9.5)
ASMI (male), kg/m^2^	9.9 (8.8–10.9)
ASMI (female), kg/m^2^	7.9 (7.3–8.4)
Fat mass, kg	38 (33.2–44.4)
Fat mass (male), kg	34.8 (30.2–41.3)
Fat mass (female), kg	40.9 (34.5–48.5)
Percentage of body fat, %	43.1 (36.2–47.8)
Percentage of body fat (male), %	35.1 (31.1–36.7)
Percentage of body fat (female), %	46.7 (43.5–50.4)
Visceral adipose tissue mass, kg	2.1 (1.5–2.6)
Visceral adipose tissue mass (male), kg	2.4 (2.2–2.9)
Visceral adipose tissue mass (female), kg	1.7 (1.3–2.4)
Subcutaneous adipose tissue mass, kg	2.3 (1.7–2.9)
Subcutaneous adipose tissue mass (male), kg	1.7 (1.6–2.7)
Subcutaneous adipose tissue mass (female), kg	2.6 (1.9–2.9)
ASMM, kg	44.7 (39–59.8)
ASMM (male), kg	32.1 (29.4–34.5)
ASMM (female), kg	20.3 (19.3–22.3)
ASMM/W (male), %	30.5 (29.1–31.9)
ASMM/W (female), %	24.1 (22.6–25.1)

Note: The presented values represent the median (interquartile range); ASMI, appendicular skeletal muscle mass index; ASMM, appendicular skeletal muscle mass; ASMM/W, appendicular skeletal muscle mass to body weight ratio.

**Table 4:** Correlation between SMM, skeletal muscle indices, FM, and %BF in patients with MASLD and HTN depending on gender (presented in the form of Spearman’s rank correlation coefficients).

		Parameters Measured by DEXA			
		FM	%BF	SMM	ASMM/W
**Parameters Measured by BIA**	FM	0.833 ** (m) 0.851 ** (f)			
	%BF		0.482 ** (m)0.577 ** (f)		
	SMM			0.818 ** (m)0.533 ** (f)	
	SMM/W				0.609 ** (m)0.626 ** (f)

Note: ** *p* < 0.01, *p*-value denotes statistical significance of correlation; (m): Spearman’s rank correlation coefficients for male patients; (f): Spearman’s rank correlation coefficients. ASMI, appendicular skeletal muscle mass index; ASMM, appendicular skeletal muscle mass; ASMM/W, appendicular skeletal muscle mass to body weight ratio; %BF, body fat percentage; BIA, bioimpedance analysis; DEXA, dual-energy X-ray absorptiometry; FM, fat mass; SMM, skeletal muscle mass; SMM/W, skeletal muscle mass to body weight ratio.

## Data Availability

The data presented in this study are available on reasonable request from the corresponding author. The data are not publicly available due to privacy restrictions.

## References

[1] Eguchi Y., Hyogo H., Ono M., Mizuta T., Ono N., Fujimoto K., Chayama K., Saibara T., JSG-NAFLD (2012). Prevalence and Associated Metabolic Factors of Nonalcoholic Fatty Liver Disease in the General Population from 2009 to 2010 in Japan: A Multicenter Large Retrospective Study. J. Gastroenterol..

[2] Younossi Z., Aggarwal P., Shrestha I., Fernandes J., Johansen P., Augusto M., Nair S. (2022). The Burden of Non-Alcoholic Steatohepatitis: A Systematic Review of Health-Related Quality of Life and Patient-Reported Outcomes. JHEP Rep. Innov. Hepatol..

[3] Wong V.W.-S., Ekstedt M., Wong G.L.-H., Hagström H. (2023). Changing Epidemiology, Global Trends and Implications for Outcomes of NAFLD. J. Hepatol..

[4] Tkachev A.V., Tarasova G.N., Groshilin V.S., Vasilchenkov D.A., Ushakova T.I., Blinov D.V. (2016). Prevalence of nonalcoholic fatty liver disease in outpatients in Rostov-on-Don: Regional results of the DIREG-2 study. Ter. Arkhiv.

[5] Duell P.B., Welty F.K., Miller M., Chait A., Hammond G., Ahmad Z., Cohen D.E., Horton J.D., Pressman G.S., Toth P.P. (2022). Nonalcoholic Fatty Liver Disease and Cardiovascular Risk: A Scientific Statement from the American Heart Association. Arterioscler. Thromb. Vasc. Biol..

[6] Cao L., An Y., Liu H., Jiang J., Liu W., Zhou Y., Shi M., Dai W., Lv Y., Zhao Y. (2024). Global Epidemiology of Type 2 Diabetes in Patients with NAFLD or MAFLD: A Systematic Review and Meta-Analysis. BMC Med..

[7] Konyn P., Alshuwaykh O., Dennis B.B., Cholankeril G., Ahmed A., Kim D. (2023). Gallstone Disease and Its Association With Nonalcoholic Fatty Liver Disease, All-Cause and Cause-Specific Mortality. Clin. Gastroenterol. Hepatol..

[8] Byrne C.D., Targher G. (2020). NAFLD as a Driver of Chronic Kidney Disease. J. Hepatol..

[9] Ahmed M.H., Byrne C.D. (2010). Obstructive Sleep Apnea Syndrome and Fatty Liver: Association or Causal Link?. World J. Gastroenterol. WJG.

[10] Glass L.M., Hunt C.M., Fuchs M., Su G.L. (2019). Comorbidities and Nonalcoholic Fatty Liver Disease: The Chicken, the Egg, or Both?. Fed. Pract..

[11] Buss J. (2014). Limitations of Body Mass Index to Assess Body Fat. Workplace Health Saf..

[12] Wells J.C.K. (2007). Sexual Dimorphism of Body Composition. Best Pract. Res. Clin. Endocrinol. Metab..

[13] Kim G., Lee S.-E., Lee Y.-B., Jun J.E., Ahn J., Bae J.C., Jin S.-M., Hur K.Y., Jee J.H., Lee M.-K. (2018). Relationship between Relative Skeletal Muscle Mass and Nonalcoholic Fatty Liver Disease: A 7-Year Longitudinal Study. Hepatology.

[14] Cruz-Jentoft A.J., Baeyens J.P., Bauer J.M., Boirie Y., Cederholm T., Landi F., Martin F.C., Michel J.-P., Rolland Y., Schneider S.M. (2010). Sarcopenia: European Consensus on Definition and Diagnosis: Report of the European Working Group on Sarcopenia in Older People. Age Ageing.

[15] Cruz-Jentoft A.J., Bahat G., Bauer J., Boirie Y., Bruyère O., Cederholm T., Cooper C., Landi F., Rolland Y., Sayer A.A. (2019). Sarcopenia: Revised European Consensus on Definition and Diagnosis. Age Ageing.

[16] Beaudart C., McCloskey E., Bruyère O., Cesari M., Rolland Y., Rizzoli R., Araujo de Carvalho I., Amuthavalli Thiyagarajan J., Bautmans I., Bertière M.-C. (2016). Sarcopenia in Daily Practice: Assessment and Management. BMC Geriatr..

[17] Shepherd J., Ng B., Sommer M., Heymsfield S.B. (2017). Body Composition by DXA. Bone.

[18] Choi J.W., Yoo J.-J., Kim S.G., Kim Y.S. (2022). Bioelectrical Impedance Analysis Can Be an Effective Tool for Screening Fatty Liver in Patients with Suspected Liver Disease. Healthcare.

[19] Branco M.G., Mateus C., Capelas M.L., Pimenta N., Santos T., Mäkitie A., Ganhão-Arranhado S., Trabulo C., Ravasco P. (2023). Bioelectrical Impedance Analysis (BIA) for the Assessment of Body Composition in Oncology: A Scoping Review. Nutrients.

[20] Lemos T., Gallagher D. (2017). Current Body Composition Measurement Techniques. Curr. Opin. Endocrinol. Diabetes Obes..

[21] Stewart S.P., Bramley P.N., Heighton R., Green J.H., Horsman A., Losowsky M.S., Smith M.A. (1993). Estimation of Body Composition from Bioelectrical Impedance of Body Segments: Comparison with Dual-Energy X-ray Absorptiometry. Br. J. Nutr..

[22] Thomson R., Brinkworth G.D., Buckley J.D., Noakes M., Clifton P.M. (2007). Good Agreement between Bioelectrical Impedance and Dual-Energy X-ray Absorptiometry for Estimating Changes in Body Composition during Weight Loss in Overweight Young Women. Clin. Nutr..

[23] Rinella M.E., Lazarus J.V., Ratziu V., Francque S.M., Sanyal A.J., Kanwal F., Romero D., Abdelmalek M.F., Anstee Q.M., Arab J.P. (2023). A Multisociety Delphi Consensus Statement on New Fatty Liver Disease Nomenclature. Hepatology.

[24] Bedogni G., Bellentani S., Miglioli L., Masutti F., Passalacqua M., Castiglione A., Tiribelli C. (2006). The Fatty Liver Index: A Simple and Accurate Predictor of Hepatic Steatosis in the General Population. BMC Gastroenterol..

[25] Boursier J., Zarski J.-P., de Ledinghen V., Rousselet M.-C., Sturm N., Lebail B., Fouchard-Hubert I., Gallois Y., Oberti F., Bertrais S. (2013). Determination of Reliability Criteria for Liver Stiffness Evaluation by Transient Elastography. Hepatology.

[26] Fang C., Sidhu P.S. (2020). Ultrasound-Based Liver Elastography: Current Results and Future Perspectives. Abdom. Radiol..

[27] Unger T., Borghi C., Charchar F., Khan N.A., Poulter N.R., Prabhakaran D., Ramirez A., Schlaich M., Stergiou G.S., Tomaszewski M. (2020). 2020 International Society of Hypertension Global Hypertension Practice Guidelines. Hypertension.

[28] Casadei K., Kiel J. (2024). Anthropometric Measurement. StatPearls.

[29] Segal K.R., Van Loan M., Fitzgerald P.I., Hodgdon J.A., Van Itallie T.B. (1988). Lean Body Mass Estimation by Bioelectrical Impedance Analysis: A Four-Site Cross-Validation Study. Am. J. Clin. Nutr..

[30] Segal K.R., Burastero S., Chun A., Coronel P., Pierson R.N., Wang J. (1991). Estimation of Extracellular and Total Body Water by Multiple-Frequency Bioelectrical-Impedance Measurement. Am. J. Clin. Nutr..

[31] Rudnev S.G., Starunova O.A., Godina E.Z., Ivanova A.E., Zubko A.V., Starodubov V.I. (2022). The Russian Bioimpedance Database: An Update. J. Electr. Bioimpedance.

[32] Janssen I., Heymsfield S.B., Baumgartner R.N., Ross R. (2000). Estimation of Skeletal Muscle Mass by Bioelectrical Impedance Analysis. J. Appl. Physiol. 1985.

[33] Jensen B., Braun W., Geisler C., Both M., Klückmann K., Müller M.J., Bosy-Westphal A. (2019). Limitations of Fat-Free Mass for the Assessment of Muscle Mass in Obesity. Obes. Facts.

[34] Rudnev S.G., Soboleva N.P., Sterlikov S.A., Nikolaev D.V., Starunova O.A., Chernykh S.P., Eryukova T.A., Kolesnikov V.A., Melnichenko O.A., Ponomareva E.G. (2014). Bioimpedance Study of Body Composition in the Russian Population.

[35] Donini L.M., Busetto L., Bischoff S.C., Cederholm T., Ballesteros-Pomar M.D., Batsis J.A., Bauer J.M., Boirie Y., Cruz-Jentoft A.J., Dicker D. (2022). Definition and Diagnostic Criteria for Sarcopenic Obesity: ESPEN and EASO Consensus Statement. Obes. Facts.

[36] Bland M. (2015). An Introduction to Medical Statistics.

[37] Karjoo S., Auriemma A., Fraker T., Bays H.E. (2022). Nonalcoholic Fatty Liver Disease and Obesity: An Obesity Medicine Association (OMA) Clinical Practice Statement (CPS) 2022. Obes. Pillars.

[38] Misra A., Vikram N.K., Gupta R., Pandey R.M., Wasir J.S., Gupta V.P. (2006). Waist Circumference Cutoff Points and Action Levels for Asian Indians for Identification of Abdominal Obesity. Int. J. Obes. 2005.

[39] Nachit M., Kwanten W.J., Thissen J.-P., Op De Beeck B., Van Gaal L., Vonghia L., Verrijken A., Driessen A., Horsmans Y., Francque S. (2021). Muscle Fat Content Is Strongly Associated with NASH: A Longitudinal Study in Patients with Morbid Obesity. J. Hepatol..

[40] Newton J.L., Jones D.E.J., Henderson E., Kane L., Wilton K., Burt A.D., Day C.P. (2008). Fatigue in Non-Alcoholic Fatty Liver Disease (NAFLD) Is Significant and Associates with Inactivity and Excessive Daytime Sleepiness but Not with Liver Disease Severity or Insulin Resistance. Gut.

[41] Maak S., Norheim F., Drevon C.A., Erickson H.P. (2021). Progress and Challenges in the Biology of FNDC5 and Irisin. Endocr. Rev..

[42] Moreno-Navarrete J.M., Ortega F., Serrano M., Guerra E., Pardo G., Tinahones F., Ricart W., Fernández-Real J.M. (2013). Irisin Is Expressed and Produced by Human Muscle and Adipose Tissue in Association with Obesity and Insulin Resistance. J. Clin. Endocrinol. Metab..

[43] Sizoo D., de Heide L.J.M., Emous M., van Zutphen T., Navis G., van Beek A.P. (2021). Measuring Muscle Mass and Strength in Obesity: A Review of Various Methods. Obes. Surg..

[44] Mainous A.G., Rooks B.J., Medley J.F., Dickmann S.B. (2022). Body Composition among Adults at a Healthy Body Mass Index and Association with Undetected Non-Alcoholic Fatty Liver. Int. J. Obes..

[45] Ariya M., Koohpayeh F., Ghaemi A., Osati S., Davoodi S.H., Razzaz J.M., Javedan G., Ehrampoush E., Homayounfar R. (2021). Assessment of the Association between Body Composition and Risk of Non-Alcoholic Fatty Liver. PLoS ONE.

[46] Zhang Y., Xiang L., Qi F., Cao Y., Zhang W., Lv T., Zhou X. (2024). The Metabolic Profiles and Body Composition of Non-Obese Metabolic Associated Fatty Liver Disease. Front. Endocrinol..

[47] Achamrah N., Colange G., Delay J., Rimbert A., Folope V., Petit A., Grigioni S., Déchelotte P., Coëffier M. (2018). Comparison of Body Composition Assessment by DXA and BIA According to the Body Mass Index: A Retrospective Study on 3655 Measures. PLoS ONE.

[48] Vassilev G., Hasenberg T., Krammer J., Kienle P., Ronellenfitsch U., Otto M. (2017). The Phase Angle of the Bioelectrical Impedance Analysis as Predictor of Post-Bariatric Weight Loss Outcome. Obes. Surg..

[49] Yamada Y., Yoshida T., Murakami H., Kawakami R., Gando Y., Ohno H., Tanisawa K., Konishi K., Julien T., Kondo E. (2022). Phase Angle Obtained via Bioelectrical Impedance Analysis and Objectively Measured Physical Activity or Exercise Habits. Sci. Rep..

[50] Otsuka Y., Yamada Y., Maeda A., Izumo T., Rogi T., Shibata H., Fukuda M., Arimitsu T., Miyamoto N., Hashimoto T. (2022). Effects of Resistance Training Intensity on Muscle Quantity/Quality in Middle-Aged and Older People: A Randomized Controlled Trial. J. Cachexia Sarcopenia Muscle.

[51] Guerrini A., Siotto M., Germanotta M., Schirru M., Pavan A., Cipollini V., Insalaco S., Aprile I. (2023). Body Cell Mass from Bioelectrical Impedance Analysis in Patients with Stroke Undergoing Rehabilitation. Appl. Sci..

[52] Moonen H.P.F.X., Van Zanten A.R.H. (2021). Bioelectric Impedance Analysis for Body Composition Measurement and Other Potential Clinical Applications in Critical Illness. Curr. Opin. Crit. Care.

[53] Berzigotti A., Tsochatzis E., Boursier J., Castera L., Cazzagon N., Friedrich-Rust M., Petta S., Thiele M. (2021). EASL Clinical Practice Guidelines on Non-Invasive Tests for Evaluation of Liver Disease Severity and Prognosis—2021 Update. J. Hepatol..

